# 7,3′,4′-Trihydroxyisoflavone Ameliorates the Development of *Dermatophagoides farinae*-Induced Atopic Dermatitis in NC/Nga Mice

**DOI:** 10.1155/2013/636597

**Published:** 2013-11-13

**Authors:** Bo-Bae Kim, Jong Rhan Kim, Ji Hye Kim, Young Ah Kim, Jun Seong Park, Myeong-Hun Yeom, Hyong Joo Lee, Ki Won Lee, Nam Joo Kang

**Affiliations:** ^1^School of Food Science and Biotechnology, Kyungpook National University, Daegu 702-701, Republic of Korea; ^2^WCU Biomodulation Major, Department of Agricultural Biotechnology, Seoul National University, Seoul 151-921, Republic of Korea; ^3^Advanced Institutes of Convergence Technology, Seoul National University, Suwon 443-270, Republic of Korea; ^4^Skin Research Institute, Amorepacific Corporation R&D Center, Yongin-si, Gyeonggi-do 341-1, Republic of Korea; ^5^Research Institute of Bio Food Industry, Institute of Green Bio Science and Technology, Seoul National University, Pyeongchang 232-916, Republic of Korea

## Abstract

Atopic dermatitis is an inflammatory and chronically relapsing skin disorder that commonly occurs in children; the number of atopic dermatitis patients is increasing. The cause and mechanism of atopic dermatitis have not been defined clearly, although many studies are ongoing. Epidemiological studies suggest that soybean and its isoflavones have immunoregulatory activities. Here, we report that 7,3′,4′-trihydroxyisoflavone (7,3′,4′-THIF), a major metabolite of daidzin, effectively inhibited lipopolysaccharide (LPS)-induced nitric oxide (NO), tumor necrosis factor (TNF)-**α**, and interleukin (IL)-6 production in RAW 264.7 cells, and also reduced **β**-hexosaminidase secretion in RBL-2H3 cells. Moreover, 7,3′,4′-THIF significantly reduced scratching time, transepidermal water loss, and mast cell infiltration. It also decreased protease-activated receptor (PAR)-2 and IL-4 expression and increased filaggrin expression in skin lesions of NC/Nga mice. These results suggest that 7,3′,4′-THIF improves *Dermatophagoides farina* body extract-induced atopic dermatitis in NC/Nga mice.

## 1. Introduction

Atopic dermatitis (AD) is a chronically relapsing skin disorder that presents with severe itching and inflammation [1–3]. It commonly occurs in children and infants, and its incidence is increasing globally [3, 4]. The cause and mechanism of AD are complex and have not been researched in detail; it is thought that AD is due to various factors, including genetic, immunologic, and environmental factors and dysfunction of the skin barrier [2]. 

Levels of various inflammatory mediators, including tumor necrosis factor (TNF)-*α*, nitric oxide (NO), and IL-6, are high in the skin of patients with AD [5]. TNF-*α*, NO, and IL-6 play important roles in inflammatory responses [5]. When allergens invade an AD skin lesion, invading allergen binds to specific IgE on the surface of mast cells or basophils and activates them to secrete substances such as histamine that are associated with allergic reactions [6]. Additionally, *β*-hexosaminidase is secreted from mast cells together with histamine and can be a marker of mast cell activation [7].

The skin barrier dysfunction is one of the major characteristics in AD patients [8]. PAR-2 is known to be involved in epidermal permeability barrier function homeostasis [9], and its activation was mediated by protease in mite allergens which can contribute to delay of epidermal barrier recovery [10]. Therefore, increased PAR-2 activity or expression was suggested as a pathophysiological factor for AD [11]. Filaggrin is a filament aggregating protein that binds to keratin fibers in epithelial cells and its degradation products such as free amino acids, PCA, and UCA are essential for skin moisture [12]. The loss-of-function variants of filaggrin are other major risk factors for AD [13–15]. There are significant increases in the expression of IL-4, IL-5, and IL-13 mRNA and protein in acute AD lesions, suggesting the accumulation of Th2 cells [16]. IL-4, one of Th2 cytokines, plays an important role in the development of Th2 cells [17, 18], and previous studies indicated that IL-4 is a central mediator of allergic inflammation [19, 20].

Epidemiological studies suggest that soy isoflavones, popular components of soybeans, enhanced immunity [21–24]. Daidzin and genistin are the major isoflavones found in soybeans [25]. The isoflavone glucoside (daidzin and genistin) must be hydrolyzed to aglycones (daidzein and genistein) by intestinal microorganisms to be absorbed [26]. Some previous studies suggest that genistein, a metabolite of genistin, inhibits the development of AD in NC/Nga mice [27]. Daidzin and its metabolites also show numerous benefits [28, 29]; however, there have been no reports of their possible anti-AD effects. Daidzein, a metabolite of daidzin, is further metabolized to two major components, 6,7,4′-trihydroxyisoflavone (6,7,4′-THIF) and 7,3′,4′-THIF, by the enzyme cytochrome P450 1A2 (CYP1A2) [30, 31]. Here, we investigated the antiatopic dermatitis effects of daidzin and 7,3′,4′-THIF *in vitro* or *in vivo* system.

## 2. Materials and Methods

### 2.1. Chemicals and Reagents

7,3′,4′-THIF was purchased from Indofine Chemical Co., Inc. (Hillsborough, NJ, USA). Daidzin and 3,3′-diaminobenzidine tetrahydrochloride hydrate were obtained from Sigma Chemical Co. (St. Louis, MO, USA). Enzyme-linked immunosorbent assay (ELISA) kits for mouse IL-6 and mouse TNF-*α* were obtained from BD Biosciences (San Jose, CA, USA). Anti-mouse antibodies against filaggrin and PAR-2 for immunohistochemistry were purchased from Santa Cruz Biotechnology, Inc. (Dallas, Texas, USA); an antibody against IL-4 was purchased from ProSpec (EB, NJ, USA). Blocking goat serum, a secondary antibody, and an ABC kit were obtained from Vector (Burlingame, CA, USA). *Dermatophagoides farina* body extract (DFE)-AD cream was purchased from Biostir Inc. (Hiroshima, Japan).

### 2.2. Cell Culture

Mouse RAW 264.7 macrophages and rat RBL-2H3 basophilic leukemia cells were obtained from the Korean Cell Line Bank (Seoul, Republic of Korea). Dulbecco's modified Eagle's medium (DMEM), penicillin-streptomycin, and 0.5% trypsin-EDTA were obtained from GIBCO Invitrogen (Auckland, New Zeland). Fetal bovine serum (FBS) was purchased from Sigma (St. Louis, MO, USA). RAW 264.7 cells and RBL-2H3 cells were cultured in DMEM supplemented with 10% FBS and 1% penicillin/streptomycin at 37°C in a humidified atmosphere containing 5% CO_2_.

### 2.3. Measurement of NO, TNF-*α*, IL-4, and IL-6 Levels *In Vitro*

RAW 264.7 cells were seeded at a density of 5 × 10^5^ per well in a 96-well plate for NO assays and sandwich ELISA. After incubation for 24 h, the cells were stimulated with 2 *μ*g/mL LPS in the presence or absence of 7,3′,4′-THIF or daidzin for the indicated period of time. Griess reagent was used to assay NO in culture supernatants. Briefly, 100 *μ*L/well of sample was incubated at room temperature for 10 min with Griess reagent, which is a 1 : 1 mixture of 0.1% (w/v) *N*-1-naphthylethylenediamine and 1% (w/v) sulfanilamide in 5% phosphoric acid. The absorbance at 570 nm was measured. TNF-*α*, IL-4, and IL-6 levels were measured using ELISA kits according to the manufacturer's instructions. 

### 2.4. Measurement of *β*-Hexosaminidase Secretion *In Vitro*

RBL-2H3 cells were seeded at a density of 2 × 10^5^ per well in a 24-well plate for *β*-hexosaminidase assays. After incubation for 24 h, the cells were treated with 0.5 *μ*g/mL anti-DNP IgE solution (diluted in DMEM) for 2 h and then washed twice with 0.5 mL MT buffer (pH 7.3). 

MT buffer (500 *μ*L) was added in the presence or absence of 7,3′,4′-THIF or daidzin, and the cells were then incubated for 30 min at 37°C. Next, the cells were stimulated with DNP-BSA (final concentration 100 ng/mL) for 1 h and placed on ice, and the supernatants were transferred from the plate to microsample tubes. Aliquots of supernatants (50 *μ*L) were transferred to 96-well plates for ELISA analysis. A 100-*μ*L aliquot of the substrate solution was added to each supernatant well. The contents of the wells were mixed vigorously, and the plates were incubated at 37°C for 30 min. The enzymatic reaction was stopped by adding 100 *μ*L of 2 M glycine buffer. The absorbance at 405 nm was evaluated according to the manufacturer's instructions. 

### 2.5. AD Induction and Administration of 7,3′,4′-THIF

Six-week-old male NC/Nga mice were purchased from Japan SLC, Inc. (Shizuoka, Japan). The mice were housed under SPF conditions (Center for Laboratory Animal Resources, Kyungpook National University) at a temperature of 22 ± 2°C and with a 12 h/12 h light-dark cycle during the entire experiment. After 3 weeks, the mice were randomly divided into four groups (*n* = 5 per group): an untreated control group, a DFE-treated group, and groups treated with DFE plus a low or high dose of 7,3′,4′-THIF (4 or 20 mg/kg). DFE-AD cream was applied twice per week for 10 weeks (50 mg per mouse per application). To disrupt the skin barrier, 200 *μ*L of 4% (w/v) sodium dodecyl sulfate were topically applied to the shaved dorsal skin surface 3 h before DFE-AD cream application. All mice received 7,3′,4′-THIF or vehicle (2% DMSO/PBS) orally five times per week for 6 weeks.

All experiments with mice were performed in accordance with the regulations and approval of the Institutional Animal Care and Use Committee at Kyungpook National University. 

### 2.6. Evaluation of Clinical Symptoms

Scratching time was observed for 20 min per mouse to evaluate itching severity. Ear thickness was measured with vernier calipers. Transepidermal water loss (TEWL) was measured using a Tewameter TM300 device (Courage + Khazaka Electronic GmbH, Germany) according to the manufacturer's instructions.

### 2.7. Histopathological Examination

Dorsal skin lesions from each mouse were resected and fixed in 10% neutral-buffered formalin. Paraffin-embedded skin samples were sectioned to a thickness of 4 *μ*m and stained with hematoxylin and eosin for assessment of epidermal thickness. Toluidine blue staining was performed to determine the number of mast cells. All analyses were performed under a microscope at 100x and 400x magnification. 

### 2.8. Immunohistochemical Staining

Sections of formalin-fixed, paraffin-embedded tissue (4 *μ*m thick) were cut, mounted on glass slides, deparaffinized, and rehydrated. Antigen retrieval was performed by incubation with trypsin working solution. To eliminate endogenous peroxidases, slides were incubated in 0.3% hydrogen peroxide in methanol. They were next blocked by incubation with 5% normal goat serum for 30 min, and then incubated with primary antibodies against PAR-2, filaggrin, and IL-4 at 4°C overnight. The sections were then developed using ABC solution. The reaction was visualized with 3,3′-diaminobenzidine tetrahydrochloride hydrate solution and the sections were finally counterstained with hematoxylin.

### 2.9. Statistics

Data are expressed as means ± standard deviation (SD) or standard error of the mean (SEM), and significant differences were identified using Student's *t*-test. *P* values of <0.05, <0.01, and <0.001 were used as criteria for statistical significance. 

## 3. Results

### 3.1. 7,3′,4′-THIF Inhibited LPS-Induced Cytokine Production in RAW 264.7 Cells and Anti-DNP IgE-Induced *β*-Hexosaminidase Production in RBL-2H3 Cells

**Figure 1:**
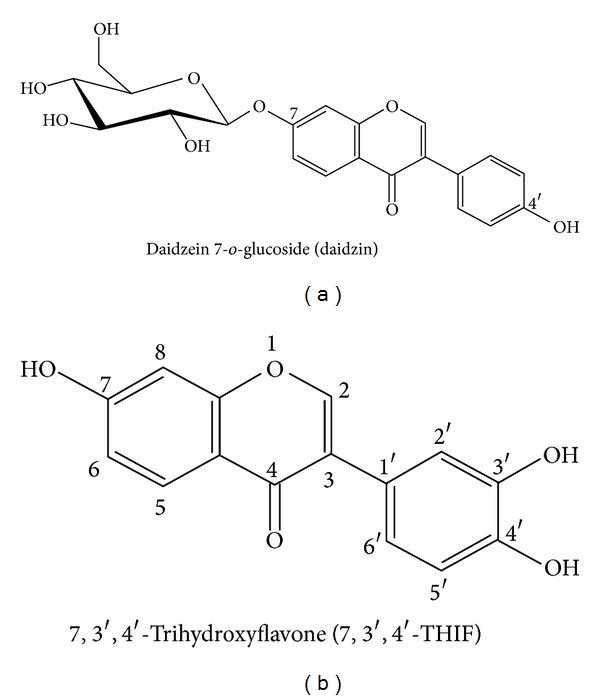
The chemical structures of (a) daidzin and (b) 7,3′,4′-THIF.

**Figure 2:**
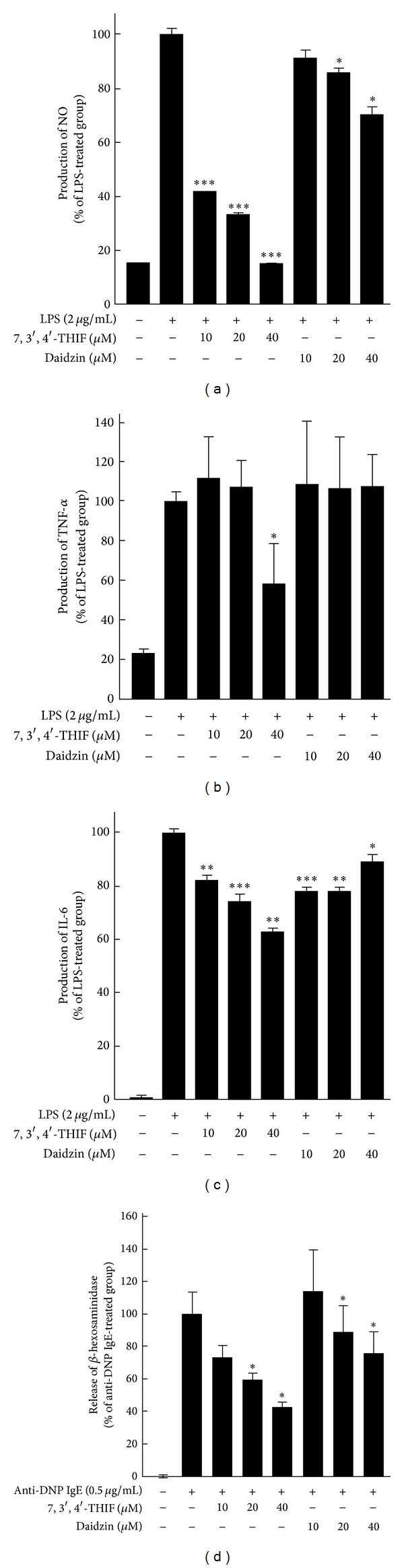
Effects of 7,3′,4′-THIF and daidzin on inflammatory cytokine and *β*-hexosaminidase production. ((a), (b), and (c)) The inhibitory effects of 7,3′,4′-THIF and daidzin on LPS-induced NO, TNF-*α*, and IL-6 production in RAW 264.7 cells. The cells were treated simultaneously with 2 *μ*g/mL LPS and samples (10, 20, or 40 *μ*M). Cytokine levels were determined by ELISA. (d) The inhibitory effects of 7,3′,4′-THIF and daidzin on anti-DNP IgE-induced *β*-hexosaminidase production in RBL-2H3 cells. The cells were treated simultaneously with 0.5 *μ*g/mL anti-DNP IgE solution and samples (10, 20, or 40 *μ*M). Data represent means ± SD (*n* = 5). *P < 0.05, **P < 0.01, and ***P < 0.001 compared with LPS- or anti-DNP IgE-treated cells.

Because various inflammatory cytokines are known to mediate inflammation and are highly expressed in AD patients [5], we first assessed the effects of 7,3′,4′-THIF (Figure 1(b)) and daidzin (Figure 1(a)) on LPS-induced NO, TNF-*α*, and IL-6 production in RAW 264.7 cells. 7,3′,4′-THIF dose-dependently inhibited the production of NO, whereas the same concentrations of daidzin had little effect (Figure 2(a)). Moreover, treatment with 7,3′,4′-THIF (40 *μ*M), but not daidzin, inhibited LPS-induced TNF-*α* production (by 41.5%) (Figure 2(b)). Similar to above data, 40 *μ*M 7,3′,4′-THIF reduced LPS-induced IL-6 production by 36.4%; 40 *μ*M daidzin caused 10.7% inhibition (Figure 2(c)). Previous studies suggested that the secretion of *β*-hexosaminidase by mast cells is a marker of mast cell activation, which is associated with allergic reactions [6, 7]. Therefore, we next investigated the release of *β*-hexosaminidase from RBL-2H3 cells. The results show that 7,3′,4′-THIF significantly and dose-dependently reduced the secretion of *β*-hexosaminidase, whereas daidzin only slightly reduced *β*-hexosaminidase secretion (Figure 2(d)). These results suggest that 7,3′,4′-THIF has more potential than daidzin to suppress skin inflammation and mast cell activation.

### 3.2. 7,3′,4′-THIF Reduced the Clinical Symptoms in NC/Nga Mice

**Figure 3:**
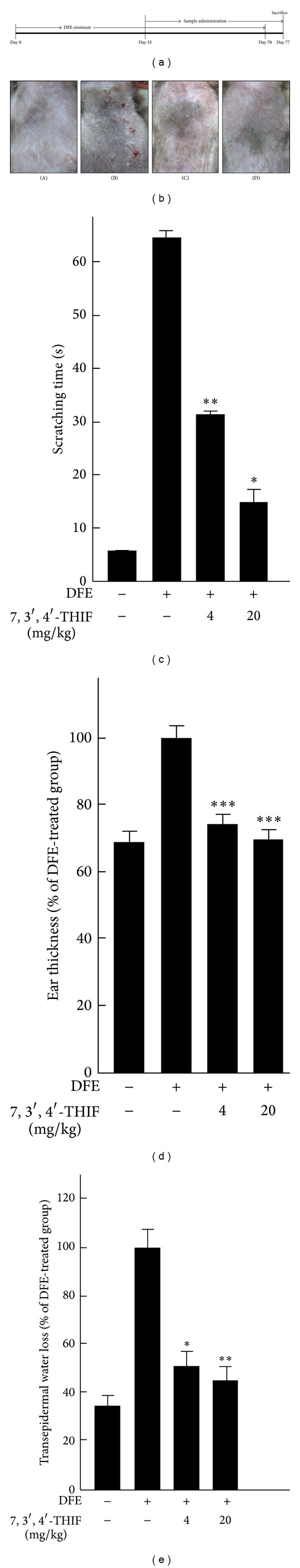
Effect of 7,3′,4′-THIF on clinical severity in NC/Nga mice. (a) The schedule of animal experiments. (b) Effect of 7,3′,4′-THIF on DFE-induced AD in NC/Nga mice. (A) Untreated control group; (B) DFE-treated group; (C) DFE plus low-dose 7,3′,4′-THIF (4 mg/kg); (D) DFE plus high-dose 7,3′,4′-THIF (20 mg/kg). (c) Effect of 7,3′,4′-THIF on scratching incidence. (d) Effect of 7,3′,4′-THIF on ear thickness. (e) Effect of 7,3′,4′-THIF on TEWL. Scratching behavior, ear thickness, and TEWL were measured 1 week before mice were euthanized. Data represent means ± SEM (*n* = 5). *P < 0.05, **P < 0.01, and ***P < 0.001 compared with DFE-treated mice.

To investigate the clinical effect of 7,3′,4′-THIF on AD in NC/Nga mice, we assessed scratching time, ear thickness, and TEWL on day 70. The dorsal skin of the DFE-treated group showed AD symptoms such as erythema/hemorrhage, edema, excoriation/erosion, and scaling/dryness. The 7,3′,4′-THIF-treated groups showed dose-dependent improvement in visual features, and the high-dose-treated group showed a status similar to that of the untreated control group (Figure 3(b)). The reduction in dermatitis severity was accompanied by a reduced scratching incidence. 7,3′,4′-THIF administration greatly lowered the scratching time (31.5 ± 0.50 and 15 ± 2.00 sec) compared to the DFE-treated group (65 ± 1.00 sec) (Figure 3(c)). In addition, ear thickness was increased by DFE compared to the untreated control group. 7,3′,4′-THIF inhibited the DFE-induced increase in ear thickness, reducing ear thicknesses to levels similar to those in the untreated control group (Figure 3(d)). Moreover, these outcomes were consistent with those of TEWL. 7,3′,4′-THIF administration made the skin markedly moister compared to the DFE-treated group (Figure 3(e)). Treatment with low and high doses of 7,3′,4′-THIF caused 50.6 and 56.8% inhibition of DFE-induced TEWL, respectively. Overall, our results revealed that oral administration of 7,3′,4′-THIF suppressed the development of AD in NC/Nga mice.

### 3.3. 7,3′,4′-THIF Administration Reduced Epidermal Thickening and Mast Cell Infiltration in Skin Lesions

**Figure 4:**
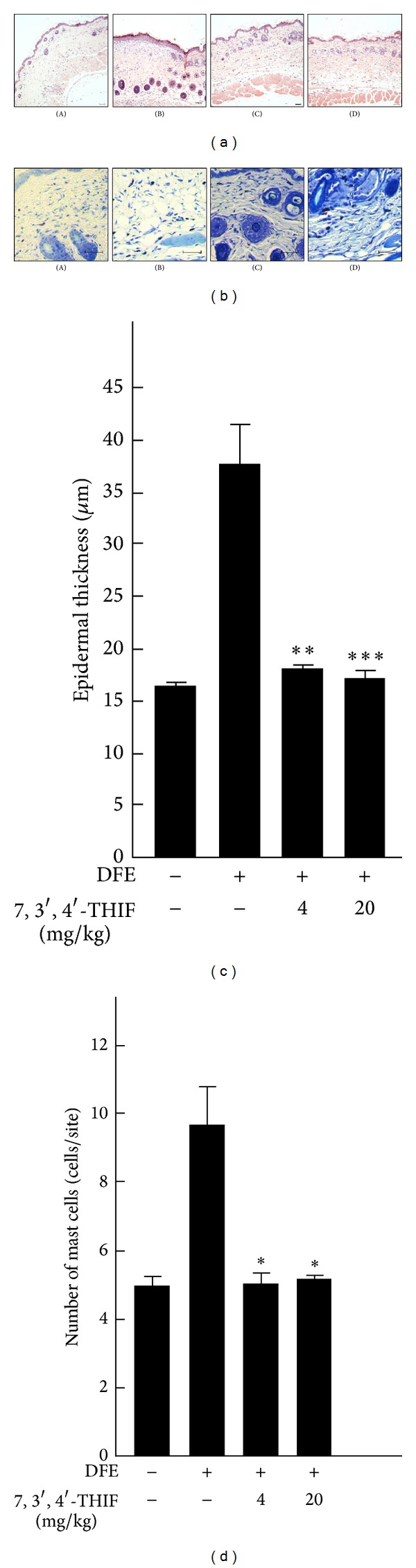
Histological features of AD skin lesions in NC/Nga mice. (a) Hematoxylin and eosin staining features of skin lesions. Epidermal hyperplasia was evaluated in randomized three sites under a microscope at 100x magnification. Scale bar: 50 *μ*m. (b) Toluidine blue staining for assessment of mast cell infiltration in skin lesions. Mast cells were counted in three random 0.025 mm^2^ sites under a microscope at 400x magnification. Scale bar: 50 *μ*m. Data represent means ± SEM (*n* = 5). *P < 0.05, **P < 0.01, and ***P < 0.001 compared with DFE-treated mice. (A) Untreated control group; (B) DFE-treated group; (C) DFE plus low-dose 7,3′,4′-THIF (4 mg/kg); (D) DFE plus high-dose 7,3′,4′-THIF (20 mg/kg).

According to a previous study, the skin lesions of AD patients show characteristic features, including epidermal thickening and deepening into the upper dermis, in which mast cells were highly accumulated [32]. On day 77, all mice were killed and their dorsal skin was resected. The improvement of clinical features by 7,3′,4′-THIF was also confirmed by analysis of hematoxylin and eosin staining and toluidine blue staining. The DFE-treated group (37.3 ± 3.95 mm) exhibited an almost 2.5-fold increase in epidermal thickness compared to the untreated control group (16.1 ± 0.39 mm) (Figures 4(a) and 4(c)). A low or high dose of 7,3′,4′-THIF reduced the epidermis thickness to a level comparable to that in the untreated control group (17.7 ± 0.50 mm and 16.7 ± 0.83 mm). The infiltration of mast cells was also increased in the DFE-treated group (9.7 ± 1.10 cells per site), and 7,3′,4′-THIF decreased the number of mast cells (5.3 ± 0.46 and 4.9 ± 0.24 cells per site) to a level similar to that in the untreated control group (4.9 ± 0.25 cells per site) (Figures 4(b) and 4(d)). These results indicate that oral administration of 7,3′,4′-THIF suppressed DFE-induced AD-like skin lesions in NC/Nga mice by reducing epidermal thickness and the infiltration of mast cells.

### 3.4. 7,3′,4′-THIF Regulated PAR-2, Filaggrin, and Th2 Cytokine (IL-4) Expression in Skin Lesions in NC/Nga Mice

**Figure 5:**
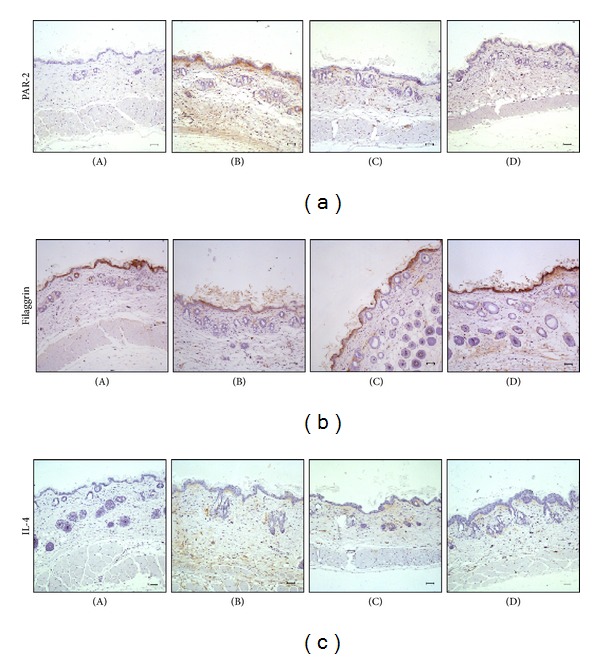
Effect of 7,3′,4′-THIF on the expression of PAR-2, filaggrin, and IL-4 in skin lesions. Immunohistochemical analysis of (a) PAR-2, (b) filaggrin, and (c) IL-4. Skin lesions were evaluated under a microscope at 100x magnification. Scale bar: 50 *μ*m. (A) Untreated control group; (B) DFE-treated group; (C) DFE plus low-dose 7,3′,4′-THIF (4 mg/kg); (D) DFE plus high-dose 7,3′,4′-THIF (20 mg/kg).

Recent studies revealed that aberrant expression and activation of PAR-2 and filaggrin mutations play crucial roles in the abnormal skin barrier function and skin lesions in AD patients [13]. Moreover, the skin barrier defect in AD is aggravated by atopic immune responses, including immune deviation *versus* Th2 dominance and increased IgE production [33]. The Th2 cytokine IL-4 is known to contribute to the development of Th2 cells and is overexpressed in AD-affected skin [16–18]. Therefore, we performed immunohistochemical analysis of PAR-2, filaggrin, and IL-4 expression to determine the effects of 7,3′,4′-THIF. As shown in Figures 5(a) and 5(c), PAR-2 and IL-4 were highly expressed in the dermis in AD skin lesions in the DFE-treated group. Interestingly, 7,3′,4′-THIF significantly decreased the levels of PAR-2 and IL-4. Filaggrin expression was reduced in the DFE-treated group, while 7,3′,4′-THIF administration restored the expression of filaggrin (Figure 5(b)). Thus, by modulating PAR-2, filaggrin, and IL-4 expressions, 7,3′,4′-THIF might serve as a highly effective agent for improving AD-like skin lesions.

## 4. Discussion

Isoflavones are most abundant in soybeans, and are present in appreciable concentrations in various other beans, legumes, sprouts, clover, and alfalfa [28, 34]. In plants, isoflavones are mostly present as glucosides (daidzin and genistin). On ingestion, aglycones (daidzein and genistein) are liberated from glucosides following metabolism by intestinal microorganisms [34, 35]. These aglycones are absorbed more rapidly and in higher amounts than the corresponding glucosides in humans [36]. Several studies have shown that soybean extract and isoflavones have antiallergic effects [21, 22, 24]. The major aglycones daidzein and genistein were investigated to determine the impact of the Th2-polarized responses on allergic rhinitis in 1,002 pregnant women. The results suggest that consuming soy products abundant in isoflavones (tofu and cooked soybeans) significantly reduced symptoms of allergic rhinitis [23]. 7,3′,4′-THIF is a major metabolite of daidzein that is produced by hydroxylation of its 3′ carbon. Genistein was reported to have an anti-AD effect in NC/Nga mice [27], but daidzein and 7,3′,4′-THIF have not been investigated in a closed AD model. According to our results, 7,3′,4′-THIF had stronger inhibitory effects on the production of inflammatory cytokines and *β*-hexosaminidase. Based on this result, we suggest that 7,3′,4′-THIF inhibits skin inflammation and mast cell activation. 

In this study, we demonstrated the effects of 7,3′,4′-THIF on AD using a DFE-induced AD model. 7,3′,4′-THIF improved visual clinical features, and reduced ear thickening and scratching behavior. It also inhibited TEWL. Furthermore, orally administered 7,3′,4′-THIF significantly inhibited epidermal thickening and infiltration of mast cells. The improved statuses were similar to those in the untreated control group. 

Invasion of external allergens is an important risk factor for the development of AD. Allergens can invade the skin more easily if the skin barrier is disrupted. As previously mentioned, PAR-2 and filaggrin have a crucial function in maintaining epidermal barrier homeostasis [37]. Levels of the endogenous PAR-2 agonist tryptase were increased up to fourfold in skin lesions of AD patients [10]. Topical application of PAR-2 activator peptides to affected skin was reported to delay barrier recovery [11]. PAR-2 is also related to itch, and its levels on primary afferent nerve fibers in skin biopsies from AD patients were markedly increased [10]. PAR-2 is expressed during immunologic reactions and it helps dendritic cells (DCs) to mature [13, 38]. Intracutaneous injection of PAR-2 agonists provoked increased and prolonged itch, and the PAR-2-mediated itch pathway provides a link to therapies for pruritus [10].

Filaggrin is a filament aggregating protein that binds to keratin fibers in epithelial cells. It aggregates the keratin filaments into tight bundles and its degradation products such as free amino acids, PCA, and UCA are essential for skin moisture [13]. According to our results, 7,3′,4′-THIF suppressed PAR-2 expression and increased the expression of filaggrin. 7,3′,4′-THIF relieves itch and maintains skin barrier moisture. AD is provoked by an imbalance between the Th1 and Th2 immune responses. Th2 cell-related responses are mediated by the Th2 cytokines IL-4, IL-5, and IL-13, whereas Th1 cell-related responses are mediated by IFN-*γ* [4, 39]. Notably, Th2 cytokines are key factors in the progression of inflammatory and immunologic skin disorders to chronic diseases such as AD. These cytokines have a strong association with the onset and aggravation of AD. Indeed, cytokine levels are increased in AD-like skin lesions. Therefore, Th2 normalization is important for relief of AD symptoms. 7,3′,4′-THIF also regulated the expression of one of Th2 cytokines, IL-4. 

In patients with AD, serum IgE levels are well correlated with disease severity [40]. In this study, IgE levels in the AD group were significantly increased compared with those in the noninduction group; however, 7,3′,4′-THIF had no effect on serum IgE level (data not shown). 

## 5. Conclusions 

We demonstrated that 7,3′,4′-THIF improved AD symptoms in DFE-treated NC/Nga mice. We expect our findings to be applicable to the improvement of AD. Consumption of soybeans, which have long been a part of the diet in Asian countries, and their ingredients have recently increased globally. Development and consuming of 7,3′,4′-THIF-rich soybeans would facilitate prevention or management of AD and other Th2-polarized diseases according to the selective needs of the individual.

## Abbreviations

AD: Atopic dermatitis
6,7,4′-THIF: 6,7,4′-trihydroxyisoflavone
7,3′,4′-THIF: 7,3′,4′-trihydroxyisoflavone
DFE: *Dermatophagoides farinae* body extract
IL: Interleukin
LPS: Lipopolysaccharides
NO: Nitric oxide
PAR-2: Protease activated receptor-2
SPF: Specific pathogen free
TNF-*α*: Tumor necrosis factor-*α*.
